# Corifollitropin alfa versus daily recombinant FSH for ovarian stimulation in women of advanced maternal age: a cost-minimization analysis in an out-of-pocket setting

**DOI:** 10.3389/frph.2026.1939114

**Published:** 2026-09-16

**Authors:** G. N. Cecchino, B. R. Carvalho, Y. Ramires, R. L. P. Bueno, L. A. Lucio, V. P. Santana

**Affiliations:** 1 Mater Prime and Mater Lab, São Paulo, Brazil; 2 Bruno Ramalho Reprodução Humana, Brasília, Brazil; 3 Faculdade de Ciências da Educação e da Saúde, Curso de Medicina, Centro Universitário de Brasília - CEUB, Brasília, Brazil; 4 Government Relations, Access and Communication, Organon Brazil; 5 Researcher at Escola Nacional de Administração Pública, Brasília, Brazil; 6 Medical Affairs, Organon Brazil

**Keywords:** assisted reproductive technology, corifollitropin alfa, cost-minimization analysis, ovarian stimulation, recombinant follitropin, drug costs, female fertility agents

## Abstract

**Background:**

In many countries, Assisted Reproductive Technology (ART) remains funded predominantly out-of-pocket, with no private insurance reimbursement and only limited public provision. For many individuals, the cost of a single ART cycle may exceed monthly income, rendering treatment financially inaccessible. Therefore, even modest differences in medication costs can meaningfully influence treatment initiation and cycle completion

**Aim:**

Given the limited regional evidence and the need for context-specific economic evaluations in Latin America, the present study aims to evaluate the economic impact of using Corifollitropin Alfa (CFA), a long-acting gonadotropin, compared with daily recombinant FSH for controlled ovarian stimulation (COS) in ART treatments in Brazilian women, under the assumption of equivalent clinical effectiveness.

**Method:**

A cost-minimization analysis was conducted from a Brazilian private-sector, out-of-pocket patient perspective, following national and international guidelines for healthcare economic evaluations. Medication reference prices, including taxes, were obtained from the official Brazilian regulatory database as of May 2025. Sensitivity analyses were performed to evaluate different gonadotropin dosing strategies in standardized COS protocols for women aged 35–42 years.

**Results:**

Across all scenarios tested, CFA was consistently associated with substantial cost reductions compared with daily rFSH. Assuming the equipotency between a single injection of CFA 150 µg and rFSH 300 IU/day for the first 7 days of COS, CFA plus follitropin alfa versus follitropin alfa alone reduced the pharmacological cost by approximately 52.1%. Similarly, CFA plus follitropin beta showed a reduction of approximately 56.3% in medication costs when compared to follitropin beta alone. These findings remained robust across all sensitivity analyses.

**Conclusion:**

CFA was associated with substantial pharmacological cost savings of more than 50% when compared to follitropin alfa or follitropin beta, assuming that a single injection of CFA 150 µg is equipotent to daily injections of rFSH 300 IU for the first 7 days of COS. Although further real-world studies are needed to validate and expand on these findings, this analysis provides meaningful evidence to support patient-centered decision-making and healthcare planning in resource-constrained settings.

## Background

According to the most recent registry from the Latin American Network of Assisted Reproduction (REDLARA), 123,265 assisted reproductive technology (ART) cycles were initiated in Latin America in 2022, resulting in approximately 26,700 estimated births. Brazil remained the largest contributor to ART activity in the region (40.6%) (1). Consistently, the most recent report from the National Embryo Production System (SisEmbrio/Anvisa) indicates that 55,971 ART cycles were performed in Brazil in 2024, corresponding to roughly 263 cycles per million inhabitants (2).

Although ART utilization is expanding globally, with an estimated annual growth of nearly 30% in initiated cycles, substantial regional disparities persist. Notably, the total number of cycles performed annually in Latin America has remained relatively stable in recent years (1, 3), and the number of cycles per million inhabitants across the region remains markedly lower than those observed in Europe and the United States (3, 4).

This striking disparity underscores persistent economic barriers and limited insurance or governmental funding mechanisms that restrict access to infertility care across most Latin American countries. With the exception of Chile, Argentina and Uruguay, few nations in the region provide consistent public coverage for infertility treatment. Consequently, high out-of-pocket costs remain a major obstacle, and private centers are responsible for the vast majority of ART cycles. As global demand for ART is expected to rise steadily in the coming decades (5), evaluating cost-related barriers and identifying cost-minimization strategies is increasingly relevant for low- and middle-income countries.

Corifollitropin alfa (CFA) provides sustained ovarian stimulation (COS), replacing seven consecutive days of treatment with a single injection, unlike traditional regimens that require daily administration. Clinical trials have demonstrated that CFA is as effective as daily recombinant follicle-stimulating hormone (rFSH); for instance, the PURSUE study demonstrated non-inferiority in the mean number of oocytes retrieved, pregnancy rates, and live birth outcomes among women aged 35–42 years, a group frequently seeking ART (6).

In Brazil, ART remains funded predominantly out-of-pocket, with no private insurance reimbursement and only limited public provision. For many individuals, the cost of a single ART cycle may exceed monthly income, rendering treatment financially inaccessible. Therefore, even modest differences in medication costs can meaningfully influence treatment initiation and cycle completion. Given the limited regional evidence and the need for context-specific economic evaluations in Latin America, the present study performs a cost-minimization analysis comparing the use of CFA with daily rFSH for COS in Brazilian couples undergoing ART, under the assumption of equivalent clinical effectiveness.

## Methods

A cost-minimization analysis (CMA) was conducted using Microsoft Excel (Microsoft Corporation, Redmond, WA, USA) to identify the least costly COS strategy when comparing CFA and daily rFSH-based regimens. This CMA considered CFA noninferiority to daily rFSH with respect to reproductive outcomes in women aged 35–42 years in the PURSUE trial (6), since this age group represents the majority of patients undergoing ART in Latin America (1). It also assumed that a single injection of CFA 150 µg is equipotent to daily 300 IU rFSH for the first seven days of COS, regardless of ovarian reserve (7).

Consistent with the cost-minimization framework, clinical and embryological outcomes were not estimated within the economic model. Equivalent clinical effectiveness between CFA and daily rFSH was assumed on the basis of previously published randomized evidence (6, 8, 9). Therefore, the modeled outcome was restricted to gonadotropin acquisition costs.

The economic analysis was conducted from a Brazilian private sector, out-of-pocket patient perspective, following the Methodological Guidelines for Economic Evaluation published by the Brazilian Ministry of Health (10), and the Consolidated Health Economic Evaluation Reporting Standards (CHEERS*)* developed by the International Society for Pharmacoeconomics and Outcomes Research (ISPOR) (11).

Medication prices were retrieved from the official price database of the Brazilian Medicines Market Regulation Chamber (CMED), using the most recent publication available as of May 2025 (12). The analysis considered the following commercial products: Elonva® 150 µg (Vetter Pharma-Fertigung GmbH & Co. KG, Ravensburg, Germany, registered by Organon Farmacêutica Ltda., Campinas, São Paulo, Brazil), Puregon® 300 IU (Vetter Pharma-Fertigung GmbH & Co. KG, Ravensburg, Germany, registered by Organon Farmacêutica Ltda., Campinas, São Paulo, Brazil), and Gonal-f® 300 IU (Merck Serono S.p. A., Bari, Italy, or Merck Serono S.A., Aubonne, Switzerland). Factory price (PF) was used as the reference unit cost. An 18% tax on the Circulation of Goods and Services (ICMS) was added to enable nationwide cost comparisons, in accordance with Brazilian tax standards. The main characteristics of the study are summarized in Table 1. Unit prices per syringe/cartridge/pen are detailed in Table 2.

**Table 1:** Main features of the study.

Feature	Characterization
Population of Interest	Adult women aged 35 to 42 undergoing OS
Perspective	Brazilian Supplementary Health System, private sector (out-of-pocket patient perspective)
Comparators	Corifollitropin alfa 150 μg for the first 7 days of OS, followed by 2 additional days of Follitropin alfa 150–450 IU/day or Follitropin beta 100–375 IU/day Follitropin alfa 150-450 IU/day for a 9-day OS Follitropin beta 100–375 IU/day for a 9-day OS
Outcomes	Pharmacological cost
Model type	Cost-minimization analysis
Time Horizon (duration)	9 days (duration of OS)
Discount rate	Not applicable
Cost sources	Publicly available national sources aligned with the payer perspective (CMED price list) + tax
Type of analysis	Deterministic sensitivity analyses

OS, Ovarian Stimulation; CMED, Chamber of Regulation of the Drug Market.

**Table 2.** Unit cost for ovarian stimulation.

Drug	Presentation	CMED FP + 18% ICMS^a^
Corifollitropin alfa	150 µg, prefilled syringe	R$ 2,265.85
Follitropin alfa	300 IU, pen	R$ 981.11
Follitropin beta	300 IU, cartridge	R$ 1,170.89

CMED, Chamber of Regulation of the Drug Market; FP, Factory Price; ICMS = Tax on Circulation of Goods and Services.

a Values adapted from the official CMED price list (12).

Sensitivity analyses tested scenarios with different combinations and doses of gonadotropins for a 9-day COS protocol, considering CFA 150 µg and the minimum and maximum doses for follitropin alfa (150–450 IU/day) and follitropin beta (100–375 IU/day) according to the Brazilian package inserts (13, 14). Therefore, the minimum and the maximum rFSH doses explored across all scenarios ranged from 100 IU/day (follitropin beta) to 450 IU/day (follitropin alfa), depending on the product-specific ranges defined in the Brazilian package inserts. The tested scenarios were created according to the three original phase III trials by which CFA-based COS was introduced (6, 8, 9).

For international readability, all costs originally calculated in Brazilian Real (BRL) are also presented in Euro (EUR). Euro values were obtained by direct currency conversion using the official exchange rate published by the Central Bank of Brazil (Banco Central do Brasil; 1 EUR = BRL 5.8023), retrieved from the Central Bank currency converter (https://www.bcb.gov.br/conversao; accessed July 27, 2026). Euro values are provided for reference only and were not adjusted for purchasing power parity or inflation.

## Results

Differences in pharmacological costs for a 9-day OS using CFA 150 µg followed by 2 additional daily doses of rFSH, based on the doses for follitropin alfa and follitropin beta specified in the Brazilian package inserts, are illustrated in Figure 1. For follitropin alfa, costs range from R$ 2,919.85 (€ 503.22) to R$ 5,208.85 (€ 897.72), while for follitropin beta, they span from R$ 3,045.85 (€ 524.94) to R$ 5,775.85 (€ 995.44). Figure 1 also presents pharmacological cost variation for a 9-day OS regimen using only daily rFSH, ranging from R$ 2,943 (€ 507.21) to R$ 13,243.50 (€ 2,282.46) for follitropin alfa, and from R$ 3,510.00 (€ 604.93) to R$ 15,795.00 (€ 2,722.20) for follitropin beta. Consequently, when using CFA-based protocols, pharmacological costs are reduced by amounts ranging from R$ 23.15 (€ 3.99) to R$ 8,034.65 (€ 1,384.74) for follitropin alfa (corresponding to daily doses of 150 IU/day and 450 IU/day, respectively), and by R$ 464.15 (€ 79.99) to R$ 10,019.15 (€ 1,726.75) for follitropin beta (corresponding to daily doses of 100 IU/day and 375 IU/day, respectively). Across all sensitivity scenarios, the explored rFSH dose range spanned from 100 IU/day (follitropin beta) to 450 IU/day (follitropin alfa), consistent with product-specific package insert limits.

**Figure 1:**
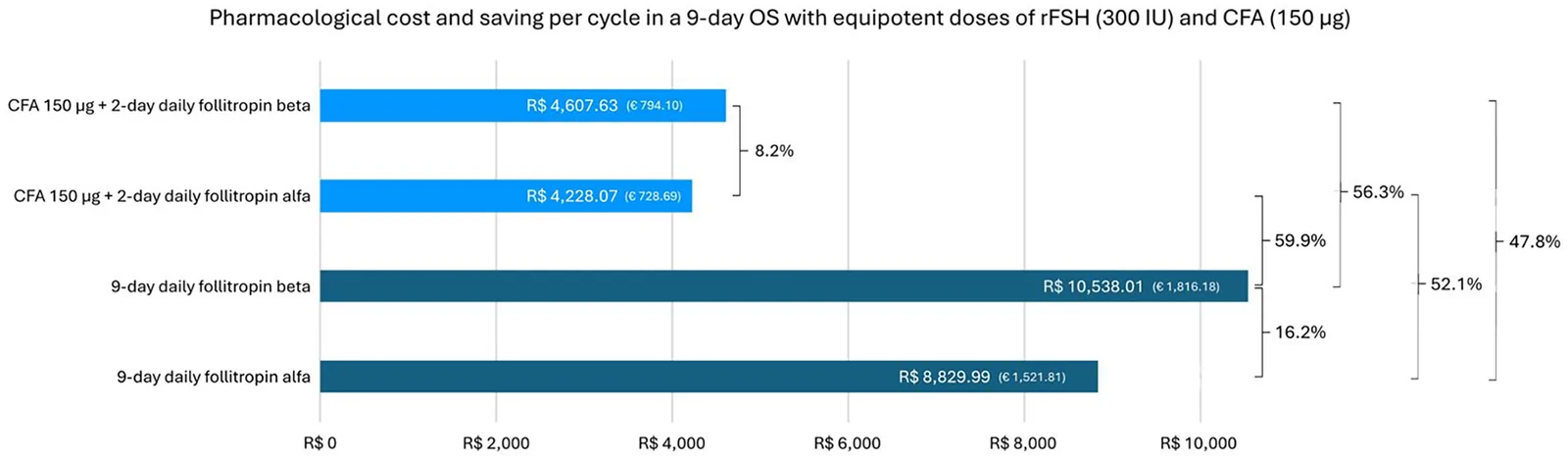
Deterministic sensitivity analysis of the total pharmacological cost per cycle for a standardized 9-day OS comparing CFA (150 µg) + 2 days of rFSH versus 9 days of daily rFSH, with rFSH doses varied within Brazilian package-insert ranges (follitropin alfa: 150–450 IU/day; follitropin beta: 100–375 IU/day). Prices for Elonva®, Gonal-f®, and Puregon® were obtained from CMED (May 2025) and increased by 18% ICMS. Euro values were obtained by direct currency conversion using the official exchange rate published by the Central Bank of Brazil (Banco Central do Brasil; 1 EUR = BRL 5.8023), retrieved from the Central Bank currency converter (https://www.bcb.gov.br/conversao; accessed July 27, 2026).

Assuming the equipotency between a single injection of CFA 150 µg and rFSH 300 IU/day for the first 7 days of OS, CFA plus follitropin alfa versus follitropin alfa alone reduced the pharmacological cost by R$ 4,601.92 (€ 793.12), indicating an expected saving of ∼52.1%. Similarly, CFA plus follitropin beta versus follitropin beta alone reduced the pharmacological cost by R$ 5,930.38 (€ 1,022.07), corresponding to a ∼56.3% reduction (Figure 2).

**Figure 2:**
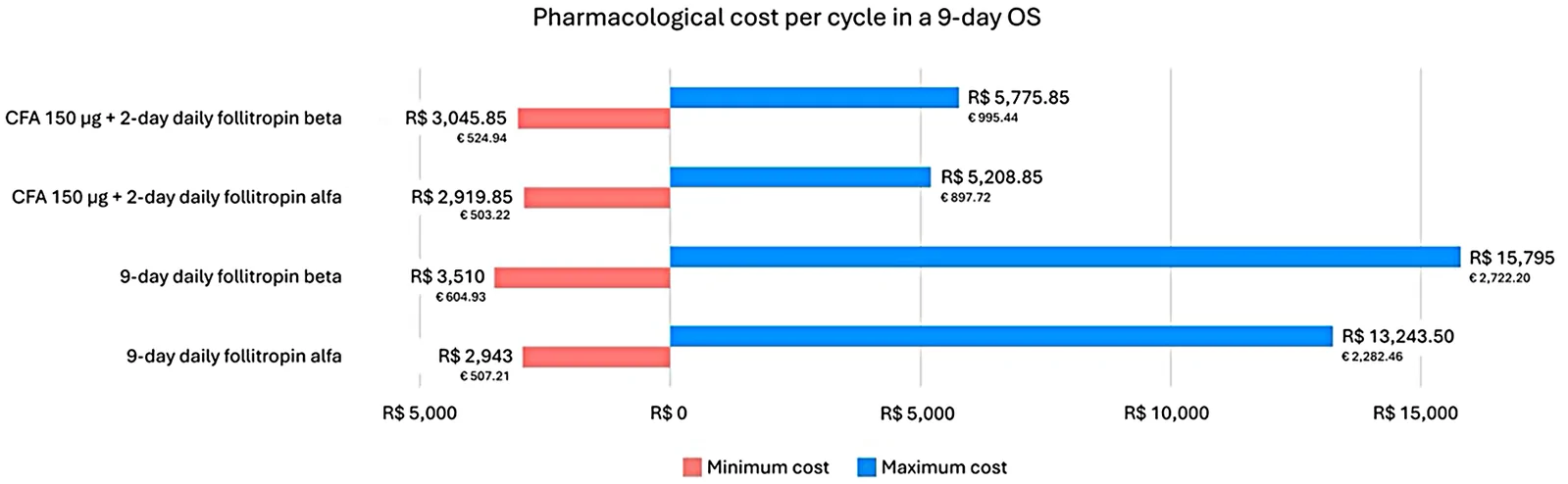
Deterministic sensitivity analysis of the total pharmacological cost per cycle for a standardized 9-day OS, assuming equipotency between CFA 150 µg and rFSH 300 IU/day for the first 7 days, followed by rFSH 300 IU/day for 2 additional days in both strategies. Brackets indicate the relative cost difference (expected savings) between protocols. Prices for Elonva®, Gonal-f®, and Puregon® were obtained from CMED (May 2025) and increased by 18% ICMS. Euro values were obtained by direct currency conversion using the official exchange rate published by the Central Bank of Brazil (Banco Central do Brasil; 1 EUR = BRL 5.8023), retrieved from the Central Bank currency converter (https://www.bcb.gov.br/conversao; accessed July 27, 2026).

## Discussion

This is the first CMA study comparing CFA to rFSH within the Brazilian context, exploring different scenarios based on the doses specified in the package inserts, and indicating that the use of a single CFA 150 µg dose for the first 7 days of a standardized 9-day OS regimen resulted in savings of ∼52% and ∼56% when compared with daily follitropin alfa and beta, respectively.

CFA is currently the only sustained follicle stimulant commercially available worldwide. While CFA clinical efficacy and safety have been well documented (6, 8, 9), real-world economic analyses are scarce and largely restricted to Spain (5, 15, 16). These studies were conducted in healthcare systems and pricing environments that differ substantially from low- and middle-income countries, including Brazil, in terms of healthcare funding, regulatory pricing, and patient payment structure, making extrapolation problematic without local validation.

From a patient-centered care perspective, prior research highlights that effectiveness remains the most valued attribute in OS decision-making, followed by cost, safety, shared decision-making, and comfort (17). This aligns with broader evidence suggesting that treatment adherence and patient satisfaction are influenced not only by clinical outcomes but also by protocol simplicity and injection burden (18, 19). In this context, CFA single-dose administration may enhance both adherence and user experience while reducing potential administration errors and the psychological toll associated with daily injections (20).

In countries like Brazil, where ART is largely financed out-of-pocket, even modest reductions in pharmacological costs may significantly impact accessibility, cycle completion, and equity. This is particularly relevant given the lack of insurance coverage and limited public provision for fertility treatments (2). Economic evaluations such as the one we present may inform value-based pricing strategies, bulk purchasing and reimbursement frameworks, especially in the context of infertility being formally recognized as a disease by the World Health Organization (21). Ultimately, our study reflects the stability of CFA's economic profile, particularly because its cost is fixed and front-loaded, whereas comparator regimens are sensitive to both duration and dose escalation. Notably, the model indicates that CFA retained a pharmacological cost advantage across the product-specific rFSH dose ranges explored in the deterministic sensitivity analyses. Importantly, the cost advantage observed here pertains specifically to gonadotropin acquisition costs; therefore, its translation to total cycle cost will depend on how medication savings compare with other cost components in each clinical setting.

Moreover, as Matorras et al. (22) proposed, the long-term societal and economic value of ART should not be overlooked, including its demographic contributions to aging populations, tax revenue generation, and improvements in quality of life for affected couples. Thus, encouraging lower-cost and patient-friendly protocols may benefit not only individual patients but also health systems seeking to balance innovation, access and financial sustainability.

This CMA has limitations. First, the model considered direct medication costs only, excluding indirect costs such as time off work, travel expenses, emotional burden, and productivity loss, factors that may be substantial in ART settings (23). Second, we did not incorporate clinical heterogeneity or OS protocol individualization, which may alter both drug use and outcomes. Third, while the model was built using official Brazilian pricing and tax structures, actual costs paid by patients may vary due to regional markups, promotional discounts, and different purchasing pathways. Finally, non-medication costs (e.g., monitoring visits, ultrasound examinations, oocyte retrieval, anesthesia, and laboratory procedures) were not included, although these components may represent a substantial proportion of total ART cycle costs. However, because both strategies were modeled under a standardized 9-day OS framework, non-medication costs would be expected to be broadly comparable between protocols and unlikely to change the direction of the findings, although small differences may occur depending on local monitoring practices.

Additionally, the analysis was based on efficacy assumptions derived from RCTs, which may not fully capture real-world variability in clinical practice. As such, further pragmatic prospective studies incorporating real-world data and broader cost dimensions, including quality-adjusted life years, are warranted. Budget impact models and cost-effectiveness studies including health system perspectives (e.g., public-private partnerships, subsidized programs, or income-based co-pays) could also offer critical insights for healthcare planners. Future analyses incorporating alternative stimulation lengths and dose adjustments guided by ovarian response may further improve the applicability of economic conclusions to individualized clinical practice.

Lastly, ART demand continues to grow globally, and new gonadotropin biosimilars are entering the market, supporting the need for future comparative studies assessing the economic impact of CFA versus FSH preparations, which may alter cost dynamics and access patterns in settings where ART is financed out-of-pocket (24). In this evolving landscape, local cost analyses should be periodically updated to reflect changes in drug pricing, availability, and clinical practice patterns.

## Conclusion

In this cost-minimization analysis, pharmacological costs differed meaningfully between a standardized 9-day OS regimen using CFA 150 µg followed by 2 days of daily rFSH and regimens using daily rFSH alone. CFA was associated with substantial pharmacological cost savings of more than 50% when compared to follitropin alfa or follitropin beta, assuming that a single injection of CFA 150 µg is equipotent to daily injections of rFSH 300 IU for the first 7 days of OS. Although further real-world studies are needed to validate and expand on these findings, this analysis provides context-specific evidence to support patient-centered decision-making and healthcare planning in resource-constrained settings. Given the high out-of-pocket burden of ART in Brazil and many other countries, such medication-level savings may translate into improved access and cycle completion, although confirmation using real-world cost and outcome data is warranted.

## Data Availability

The raw data supporting the conclusions of this article will be made available by the authors, without undue reservation.
